# Supplementary material for: Biomarkers-in-Cardiology 8 RE-VISITED—Consistent Safety of Early Discharge with a Dual Marker Strategy Combining a Normal hs-cTnT with a Normal Copeptin in Low-to-Intermediate Risk Patients with Suspected Acute Coronary Syndrome—A Secondary Analysis of the Randomized Biomarkers-in-Cardiology 8 Trial
Source: Cells. 2022 Jan 8;11(2):211. doi: 10.3390/cells11020211 (PMC8773592; doi:10.3390/cells11020211)
Supplement: Supplementary file 1 [file cells-11-00211-s001.zip › cells-1484426-supplementary.pdf]

Table S1. MACE categories.

	BIC8 Original		BIC8 RE-VISITED	
	**Standard Group (*n* = 451)**	Copeptin Group (*n* = 451)	Standard Group (*n* = 422)	Copeptin Group (*n* = 414)
MACE at 30 days	23	23	18	18
All Cause Death	2	0	2	0
Myocardial Infarction	3	8	0	5
Rehospitalisation for ACS (not ambulant)	5	2	5	2
Acute unplanned PCI	13	11	11	9
CABG	0	2	0	2

MACE: major adverse cardiac events; ACS: acute coronary syndrome; PCI: percutaneous coronary intervention; CABG: coronary artery bypass graft.

Table S2. Timing of experimental and standard serial troponin strategy.

	**All Patients (*n* = 836)**	**Standard Group (*n* = 422)**	**Copeptin Group (*n* = 414)**
Troponin 0 h (yes)	836	422	414
Troponin 3 h (yes)	384	327	57
Troponin 6 h (yes)	218	188	30
Troponin 3 h and 6 h (yes)	156	136	20
DMS eligible (Copeptin < 10 pmol/L + hs-cTnT 0 h) < 14 ng/L)	683	340	343

All patients have hs-TnT at 0h; values for Toponin at 3h and 6h refer to TnT, TnI or hs-TnT.
Abbreviation: DMS: dual marker strategy.

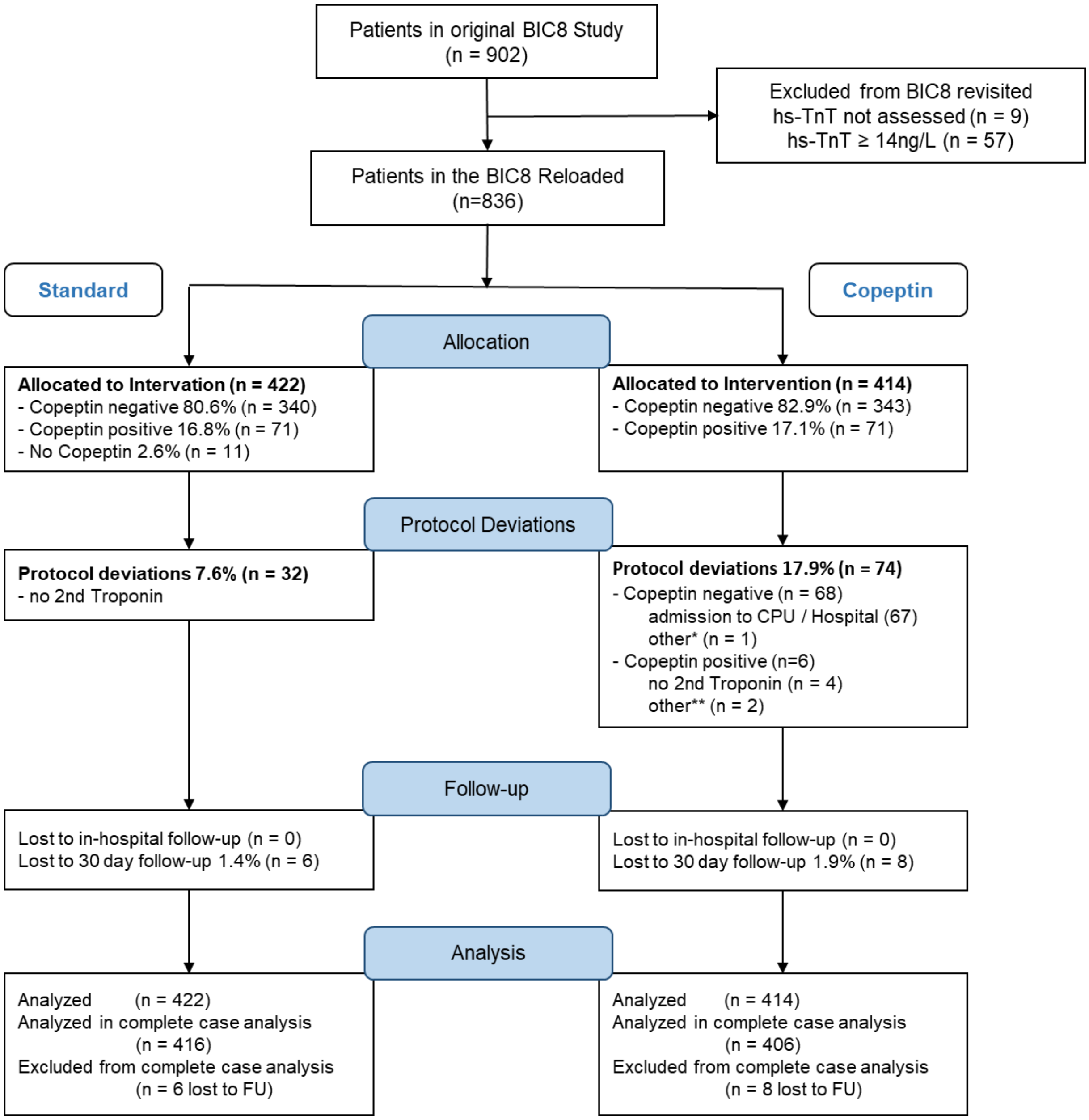

Figure S1. Consort diagram.

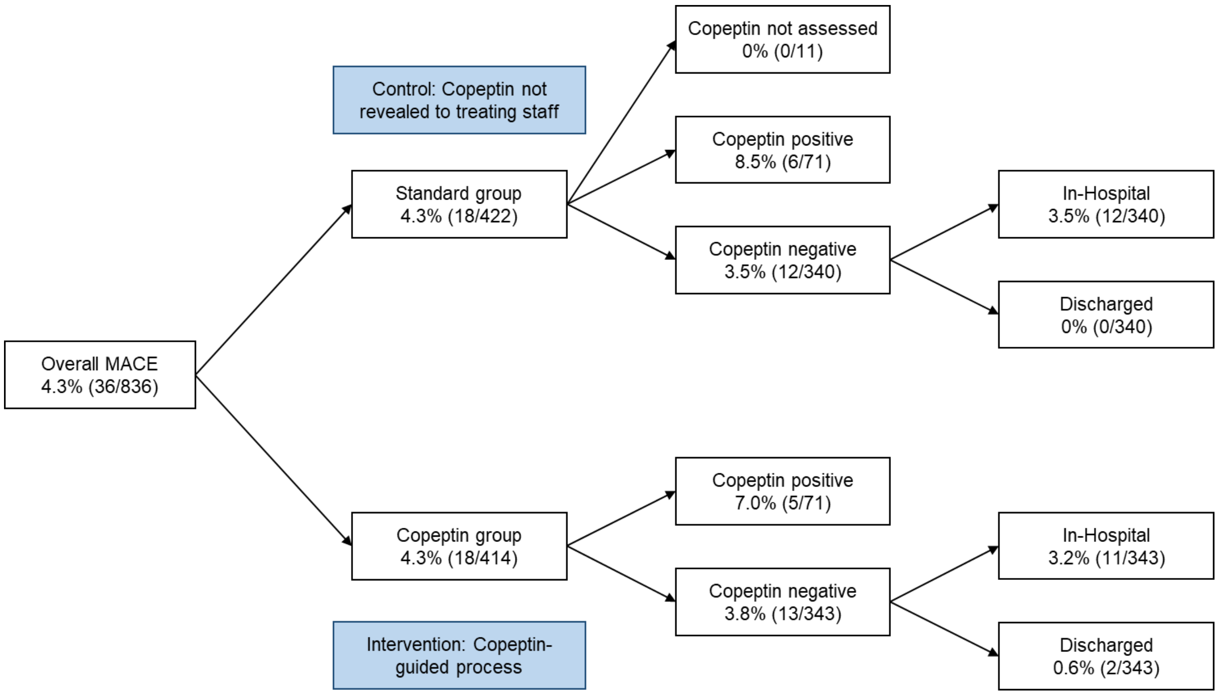

Figure S2. Major adverse cardiac events (MACE) proportions in study groups and in copeptin subgroups.
